# Finite Element Analysis of Patient-Specific 3D-Printed Cranial Implant Manufactured with PMMA and PEEK: A Mechanical Comparative Study

**DOI:** 10.3390/polym15173620

**Published:** 2023-09-01

**Authors:** Freddy P. Moncayo-Matute, Efrén Vázquez-Silva, Pablo G. Peña-Tapia, Paúl B. Torres-Jara, Diana P. Moya-Loaiza, Tony J. Viloria-Ávila

**Affiliations:** 1 Grupo de Investigación en Nuevos Materiales y Procesos de Transformación (GIMAT), Universidad Politécnica Salesiana, Sede Cuenca EC010102, Ecuador; fmoncayo@ups.edu.ec (F.P.M.-M.); ptorresj@ups.edu.ec (P.B.T.-J.); dmoyal@ups.edu.ec (D.P.M.-L.); 2 Instituto oncológico SOLCA, Sociedad de Lucha Contra el Cáncer, Cuenca EC010109, Ecuador; bioinfo@institutodelcancer.med.ec; 3 Grupo de Investigación en Biotecnología y Ambiente (INBIAM), Universidad Politécnica Salesiana, Sede Cuenca EC010102, Ecuador; tviloria@ups.edu.ec

**Keywords:** polymethylmethacrylate, polyether-ether-ketone, custom medical device, finite element analysis

## Abstract

This article reports on a patient who required a cranial protection system. Using additive manufacturing techniques and surgical planning with the help of bio-models, a patient-specific bone implant solution was proposed that allows aesthetic restoration of the affected area and provides an adequate level of protection. In addition, through a comparative analysis with finite elements, the mechanical response to external actions of the medical device, printed with two materials: polymethylmethacrylate (PMMA) and polyether-ether-ketone (PEEK), is simulated. The tested materials have recognized biocompatibility properties, but their costs on the market differ significantly. The results obtained demonstrate the similarities in the responses of both materials. It offers the possibility that low-income people can access these devices, guaranteeing adequate biomechanical safety, considering that PMMA is a much cheaper material than PEEK.

## 1. Introduction

Currently, advances are palpable in terms of the application of additive manufacturing techniques and the use of different materials and composites for medical problem solving, such as, for example, the restoration of bone structures that have suffered damage due to cancerous pathologies, or accidents of diverse nature. Among the materials that have been applied are metals and alloys, ceramics, and polymers. Precisely, among the latter are Polyether-ether-ketone (PEEK) and Polymethylmethacrylate (PMMA). These polymers have been investigated based on their biological properties (biocompatibility, osseointegration, and others), and their mechanical properties (ability to emulate natural bone behavior, responses to external loads, and so on). Regarding the good qualities of PEEK and its behavior in contact with the human body, it is possible to consult, for example, results published in [1,2,3,4,5,6,7,8,9,10,11,12,13,14,15]. These articles reported on the absence of clinical complications when PEEK was used; the improvement of the properties of this material when an adequate surface treatment was carried out; the increase in biological activity and the increase in antibacterial activity thanks to the incorporation of other material in its base matrix; how the biocompatibility improves with the modification of the microstructure of the polymer; the predominance of the Fused Deposition Modelling technique as a manufacturing method; and, in general, about the good postoperative results with patients implanted with PEEK.

In summary, PEEK is suitable for artificial bone replacements because it is biocompatible, non-toxic, and noninflammatory and osteoconductive. Its low molecular weight makes it ideal in orthopaedics for fracture fixation and osteotomies, spinal fusions, and ligament reconstructions. Its high resistance provides it with better mechanical properties compared to other traditionally used materials since it supports the loads generated in the human body. It also helps to evaluate the evolution of fractures, thanks to the fact that it is radiolucent in radiographs. Moreover, it is compatible with Computed Tomography (CT) and Magnetic Resonance Imaging (MRI) technologies, that is, PEEK does not interfere with these imaging techniques.

Before the appearance of PEEK, another polymer used in this type of surgical procedure and treatment of bone conditions was PMMA. In the review article [16], a section is dedicated to the comparison between both materials, in terms of advantages and disadvantages in implantology applications. As for PMMA, it polymerizes through an exothermic reaction that can be harmful to overlying soft tissues [17]. Implants based on this material cannot be infiltrated by new bone tissue due to its lack of porosity, it interferes with osteoconduction and vascularization, it does not interact with the surrounding tissue, and it may be susceptible to higher infection rates [18,19,20,21]. On the other hand, four studies [22,23,24,25] report similarities between PEEK and PMMA in terms of the success rate of treatment and rate of complications.

This paper reports on a planned and materialized cranioplasty with PMMA in a health institution in southern Ecuador. And as part of the investigation, the performance of the customized bone implant, manufactured with PEEK and with PMMA, is compared at the level of simulation of the mechanical response. This considers the advantage of PMMA over PEEK in terms of cost. A random internet search found the value of PMMA (https://spanish.alibaba.com/g/medical-grade-pmma.html, accessed on 1 July 2023) to be between USD 1.99 and USD 3.80 per kg. While the price of medical-grade PEEK resin (https://spanish.alibaba.com/g/medical-grade-peek.html, accessed on 1 July 2023) is between USD 1450 and USD 1470 per kg. A limitation of PEEK technology is the requirement for support structures, which incurs additional costs. Considering the above, and that the average purchasing power of the inhabitants of this area is low, the PMMA-based variant of implants remains a viable option for many people who do not have the necessary resources to afford the best PEEK-based solution. Regardless of the proven biological restrictions of PMMA (possible triggering of infectious processes caused by allergy to the material), the hypothesis that is put forward in this study is that PMMA continues to be a viable option for people whose countries of origin or residence do not provide them with a public health system that covers their needs, and whose low income prevents them from facing the high costs of PEEK as a material for therapeutic use.

## 2. Materials and Methods

### 2.1. Data Acquisition Medical Imaging and Segmentation

A computed tomography of the patient was performed, and the images were saved in a high-resolution Digital Imaging and Communications in Medicine (DICOM) file, with voxels of 512 × 512 × *Z*, where *Z* varies from 150 to 520 slices. The images were then processed using the open-source software 3D Slicer (https://www.slicer.org, accessed on 1 July 2023) to generate the Standard Tessellation Language or Stereolithography (STL) file of the anatomy of interest.

The tomographic images were segmented with a specific intensity, called Hounsfield Unit (HU), which measures the attenuation coefficient on the grey scale for compact tissues, in this case, the target anatomical region of the patient.

**Figure 1:**
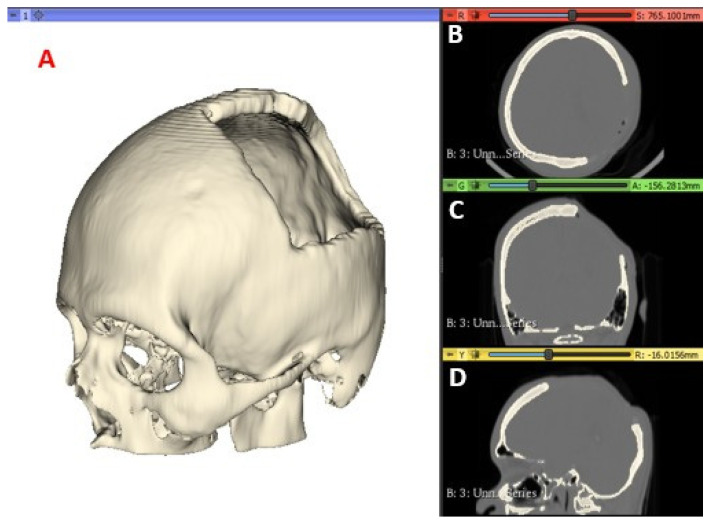
CT Scan Images: segmented model (**A**); axial view (**B**); coronal view (**C**); and sagittal view (**D**).

For the present study, values of 211.73–2755.0 HU are used to segment the cranial compact bone model. In Figure 1, the medical images and segmentation are shown.

### 2.2. Finite Element Method

#### 2.2.1. Cranial Model

To obtain the three-dimensional geometry of the patient’s cranial model, the STL file was processed by treatment of meshes and cloud of points, with reverse engineering tools provided by the Autodesk Meshmixer (https://www.meshmixer.com, accessed on 1 July 2023), and ANSYS WORKBENCH R 21.1 software (ANSYS Inc., Canonsburg, PA, USA) (Our university has the exploitation license for this software).

**Figure 2:**
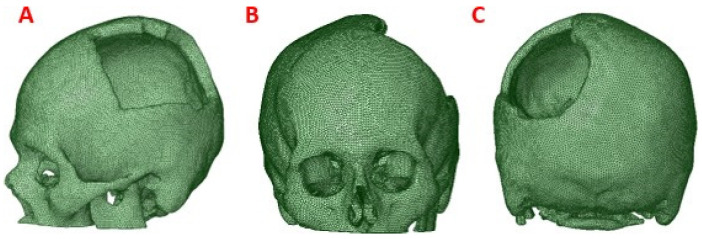
Computational model of the skull: (**A**) Lateral view, showing damage or trauma to the local parietal bone; (**B**) Front view of the cranial model, the defect is observed as well as the lack of symmetry; (**C**) Posterior view of the cranial model.

This process was applied to the anatomical model of the patient to obtain a precise 3D model of his anatomy, which was then used for finite element analysis (FEM) (Figure 2).

#### 2.2.2. Customized Implant Model

**Figure 3:**
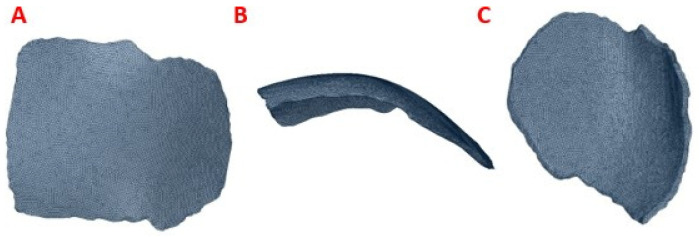
Reconstruction of the cranial 3D model with the help of the customized implant to simulate the clinical scenario: (**A**) Geometric model of the implant to cover the defect in the patient’s parietal area; (**B**) Geometric model of the customized implant, coronal contour perimeter zone; (**C**) 3D view of the implant, showing the internal shape and the variation of the angulation of the contact interface along the contour.

The post-processed model of the skull with the defect was used for the reconstruction with the cranial implant, using the Autodesk Meshmixer software 3.4. A symmetrical reference plane was created in the three-dimensional model. For the restoration of the affected area (missing cranial bone), the structural symmetry of the human body was assumed. With the editing tools of the software, the healthy side of the structure is inverted, creating a mirror image that can be superimposed on the area to be restored. Both parts of the structure are assembled to fill the cavity. Subsequently, the Boolean subtraction tool is applied to obtain the required custom implant design (Figure 3).

#### 2.2.3. Assembly of the Skull Model and Medical Device

**Figure 4:**
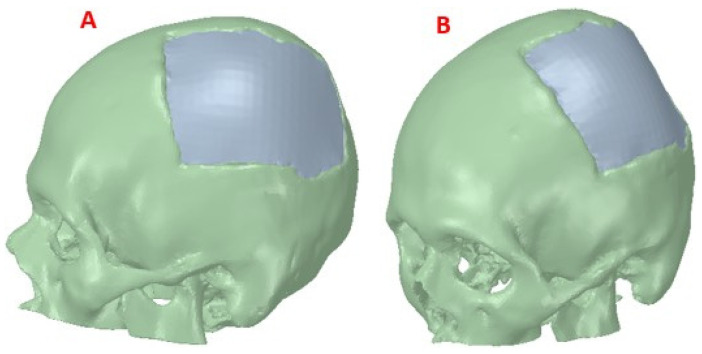
Reconstruction of the skull: (**A**) Lateral view of the skull and customized implant, showing the reconstruction; (**B**) Frontal view, defect covered by the cranial implant.

Figure 4 shows the reconstructed cranial model of the patient. The design of the implant was carried out in a personalized way, and when the person receiving the medical device requires it (in extreme cases), this model is also useful for simulations. For example, in high-performance sports activity, failures of implanted medical devices have been evidenced, causing structural and physiological damage to the bone. An adequate simulation study can contribute to the determination of the mechanical parameters that personalized medical devices must present to be able to withstand certain static loads, also simulating events that may take place during the performance of these types of activities [26].

For the simulation studies in this case, the materials used for the computational analysis were two: one of low cost, widely used in operating rooms for immediate cranioplasties, and in the dental sector, PMMA. And the second of high performance and high cost, although with better biocompatibility properties, industrial-grade PEEK, is used in Latin American countries for medical purposes.

#### 2.2.4. Coupling Details

For the successful fixation of the implant, it is important to detail the interface contours of the cranial profile and the location of the implant. For this, the perimeter area of the damage is studied as well as how the stress transfer occurs under considered external load conditions. In the article [27], the relevance of analysing the contacts of natural bone-implant interfaces is highlighted, in addition, its authors corroborate that the edges of the defect must have a surgical preparation at positive angles, while the edges of the device to be implanted must have opposite angles, in such a way that the “fit” will be the best possible, thus contributing to the coupling safety.

**Figure 5:**
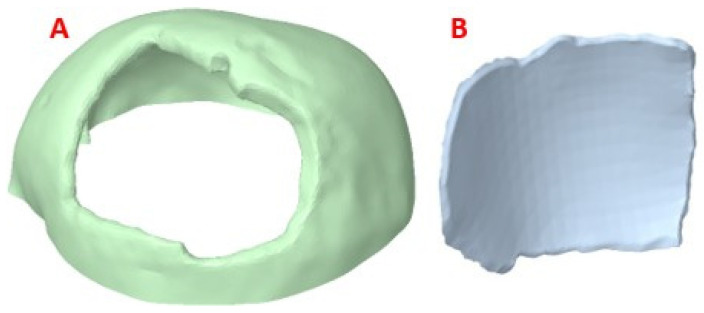
Configuration of the model to simulate the clinical scenario, highlighting the contour of the defect and adjustment of the implant: (**A**) Geometric model of the location of the defect in the left lateral parietal area of the patient; (**B**) Geometric model of the customized implant, showing the contours of the perimeter adaptation to the cranial bone.

If the implant does not have support on the cranial bone, in the presence of external loads, stresses are transferred to the fixing elements, which endangers the integrity of the system. High stresses would potentially compromise the bone morphology required for anchorage. For the case reported in this paper, the fixation systems were not analysed since full support of the implant was achieved on the cranial damage perimeter (Figure 5).

#### 2.2.5. Boundary Conditions

**Figure 6:**
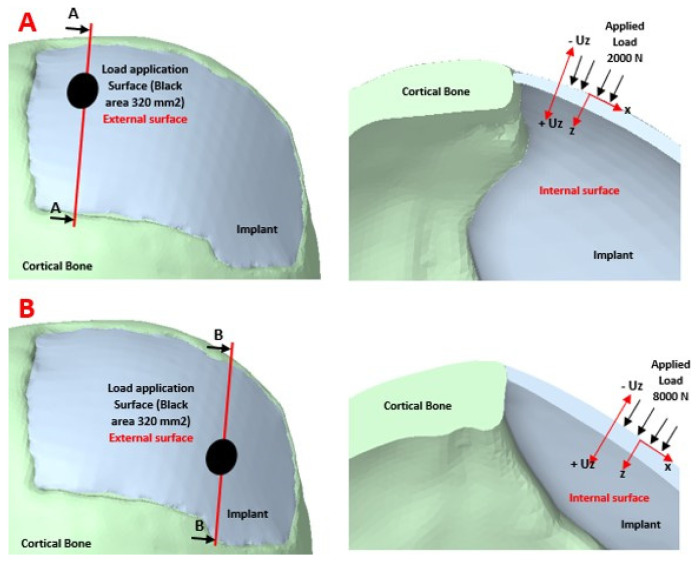
External load application scenarios: (**A**, left) Normal load of 2000 N on the implant surface in the position identified with the black circle with area of 320 mm^2^; (**A**, right) Cross section showing the effect of the applied load; (**B**, left) Normal load of 8000 *N* on the surface of the implant in the position identified with the black circle with area of 320 mm^2^; (**B**, right) Cross section showing the effect of the applied load.

The simulation study was developed with the finite element method, applied with the structural static module of the ANSYS software. The loading conditions were assigned on the external surface of the customized implant. An external load of 8000 N was applied to the lower right surface of the implant [28], which simulates the static load caused by a foreign object impact. Likewise, a load of 2000 N was applied to the upper left part of the implant, simulating the rest activity. To reduce the computational load in the simulations, the cranial model was simplified, thereby reducing the meshing and processing time. An embedment condition called “Fixed Support” was also assigned at the base of the axial slice of the skull, with zero displacements and rotations. The external charge states are observed in Figure 6.

#### 2.2.6. Mesh Conditions

**Figure 7:**
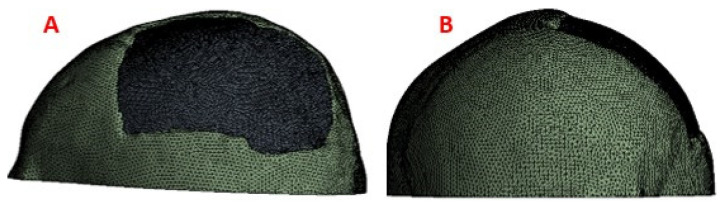
Mesh elements: (**A**) Mesh of the implant with fixation towards the skull; (**B**) The meshing of the cranial model with the implant.

The simulation was carried out with the help of the ANSYS software. The selected mesh was made up of tetrahedral elements (SOLID185). The mesh refinement tests yielded a 7% convergence guarantee. The model is composed of 329,898 nodes and 178,488 elements of size 5 mm. “Bonded contact” was also considered for border conditions at the implantcortical bone interface, the most realistic according to [29]. Details of the mesh can be seen in Figure 7.

### 2.3. Material Properties

In the computational model, the materials were assumed to be isotropic, homogeneous and linearly elastic, by [30,31]. The custom medical device was made of PMMA and PEEK. Table 1 presents the mechanical properties of each material used in the simulations.

Once the mechanical properties of the components were introduced, FEA simulations were carried out to study the behaviour of the cranial model under external static loads, which are useful to understand how the stresses are distributed in the cranial area during the performance of the considered activities.

### 2.4. Surgical Planning and Manufacturing

In the manufacture of the anatomical test model, for surgical planning, the additive manufacturing technology Fused Deposition Modelling (FDM) was applied, with the help of a Creality CR-X Pro 3D FDM printer (Shenzhen Creality 3D Technology Co., Ltd., Shenzhen, China).

As printing material, Creality’s HP PLA 1.75 mm Series filament was used. The STL file was used as the basis for reading, processing and obtaining the route code, while for the 3D printing process, the Creality Slicer 1.2.3 software was used. The support material was removed manually, paying special attention not to affect the surface of the bone structure. Table 2 presents the characteristics of the FDM technology.

**Figure 8:**
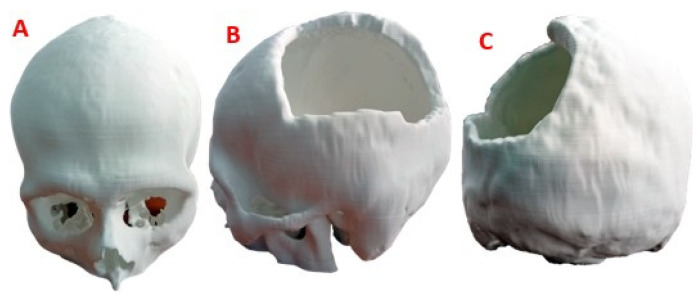
Cranial model for surgical planning: (**A**) Frontal view; (**B**) Lateral view, area of damage; (**C**) Back view.

The 3D version of the test anatomical model, printed on a 1:1 scale, can be seen in Figure 8. The level of damage in the left parietal area of the patient is considerable, so the results of the finite element analysis offer the necessary support for the medical device to withstand demand loads.

The trial anatomical model was used by the surgeon in the preoperative evaluation and surgical planning, thus verifying the functionality of the customized device.

**Figure 9:**
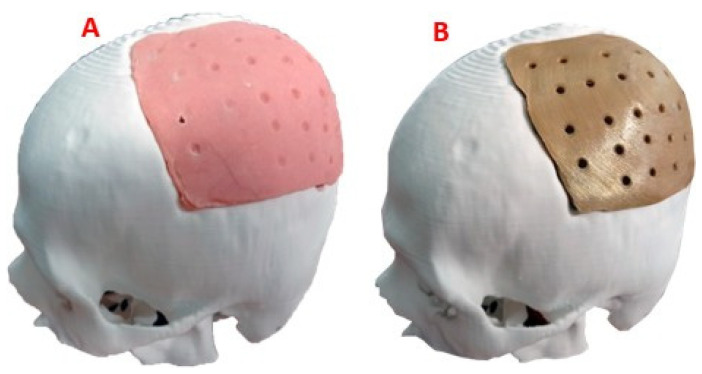
Cranial model with the patient-specific implant: (**A**) Cranial bio-model with the PMMA implant coupled; (**B**) Cranial bio-model with the PEEK implant coupled.

The digital model of the custom implant was printed in PLA. With the help of this physical model, the moulds (negative and positive) were created based on Vinyl Polysiloxane (hydrophilic material). The resulting cavities were joined to pour the PMMA solution into the free space between them, which solidifies, acquiring the shape of the final implant. The fixation holes in the final device were drilled with a surgical drill. For its part, the customized implant model, in industrial PEEK, was manufactured with the help of a FUNMAT PRO 410 printer (INTAMSYS. Shanghai, China), applying FDM additive technology. Subsequently, the definitive test of the implant was carried out on the cranial anatomical model, and it was verified that the shape was consistent with the virtual models (Figure 9).

## 3. Results

Four simulation cum-shots were performed under the “Force” load condition of 2000 N applied to the external surface of the cranial bone-coupled PMMA-based custom implant model, and with the same condition applied also on the external surface of the personalized implant model based on PEEK coupled to the cranial bone. The geometry of the personalized implants, for both materials, presents variable thicknesses. Towards the central point, where the 2000 N load was applied, the thickness is 2.58 mm; while at the point where the 8000 N load is applied, the thickness is 3.02 mm.

Likewise, for the load condition “Force” of 8000 N applied in the central zone of the external surface of the model of the personalized PMMA implant, coupled to the cranial bone. And the same load condition was applied to the central area of the external surface of the custom PEEK implant model, coupled to the cranial bone. This allowed us to obtain the VonMises stress distribution for the implant and the cranial bone, and the total deformations (Table 3).

**Figure 10:**
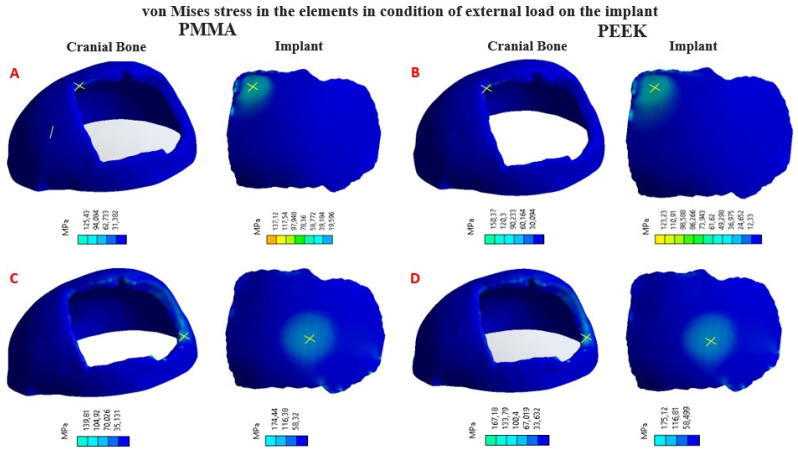
Distribution of Von Mises stresses in the implant and cranial bone (maximum values marked with a yellow X): (**A**) Load condition “Force” 2000 N, implant with PMMA; (**B**) Load condition “Force” 2000 N, implant with PEEK; (**C**) Load condition “Force” 8000 N, implant with PMMA; (**D**) Load condition “Force” 8000 N, implant with PEEK.

**Figure 11:**
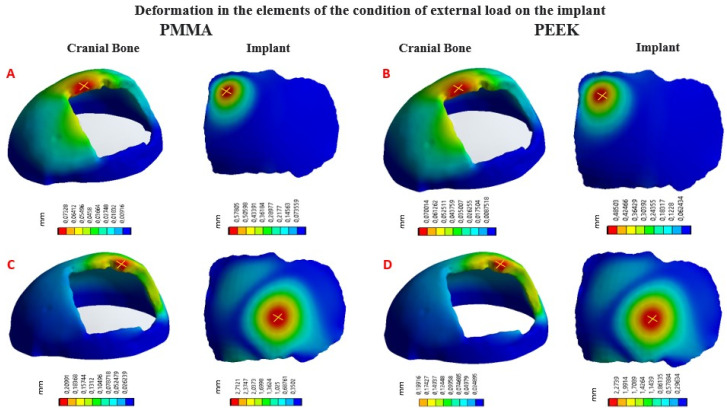
Total deformations in the implant and cranial bone (maximum values marked with a yellow X): (**A**) Load condition “Force” 2000 N, implant with PMMA; (**B**) Load condition “Force” 2000 N, implant with PEEK; (**C**) Load condition “Force” 8000 N, implant with PMMA; (**D**) Load condition “Force” 8000 N, implant with PEEK.

In Figure 10, the Von Mises stress distribution for each element can be seen, according to the state of external load. In Figure 11, the total deformations generated by the states of external loads, in the implant and the cranial bone, are detailed.

### 3.1. Statistic Analysis

**Figure 12:**
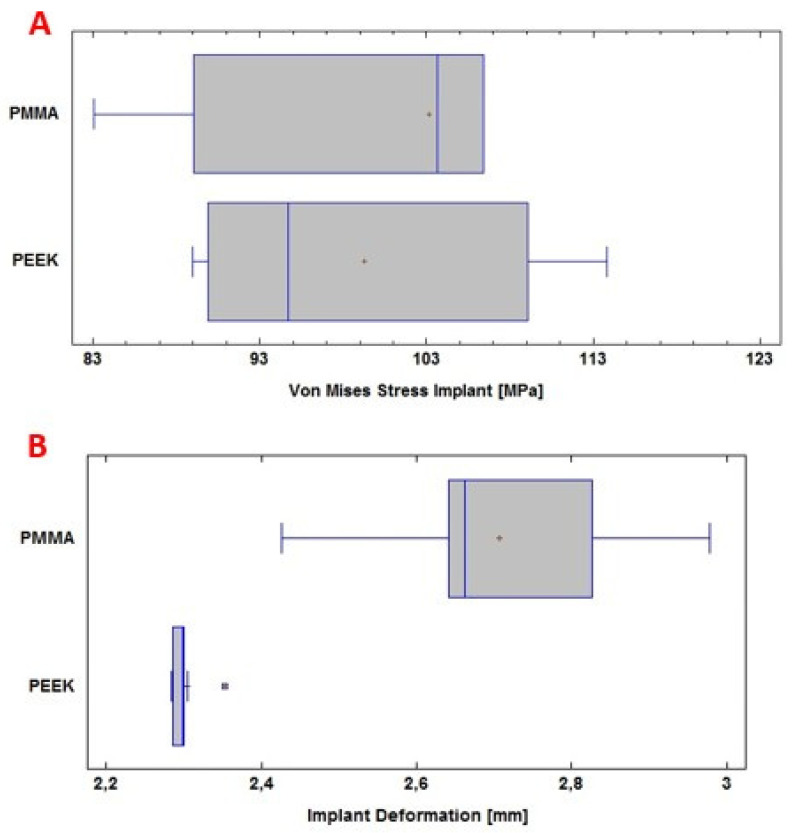
Statistical analysis: (**A**) Von Mises stresses of the PMMA and PEEK implants, respectively, on the abscissa axis, in Megapascals; (**B**) Deformation of the PMMA and PEEK implants, respectively, on the abscissa axis, in millimetres.

**Figure 13:**
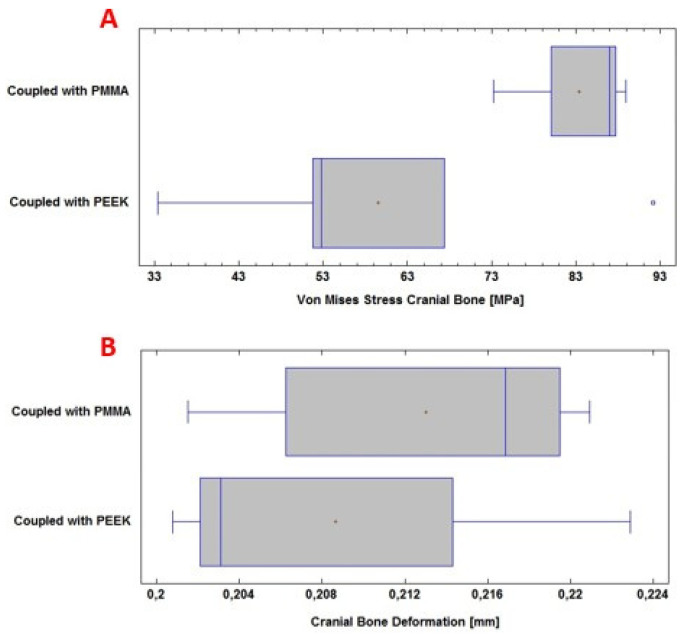
Statistical analysis: (**A**) Von Mises stresses in the cranial bone, on the abscissa axis, in Megapascals; (**B**) Deformation of the cranial bone, on the abscissa axis, in millimetres.

The analysis of the computational results was carried out by applying a comparison of means, with the help of the SPSS Statistics software 29.0 (SPSS, Inc., Chicago, IL, USA), considering that, for each simulated external load, five runs were performed with each material. The ANSYS software provides the maximum stress and deformation values. In Figure 12A (Figure 12 and Figure 13, respectively, show the box and whiskers diagrams for Von Mises stresses, and for deformations, for each material. In them, the mean values are represented by a red dot in boxes, and with respect to these points are referenced the corresponding standard deviations.), the Von Mises stress levels for the personalized implant with PMMA and PEEK are observed. Figure 12B shows the total deformation of the implant with each material under the load condition “Force” 8000 N.

In this computational study, the effect of using PMMA and PEEK for the protection of the skull against the action of a static load of 8000 N on the surface of the device was examined. The mean and standard deviation of the Von Mises stress analysis for the PMMA implant was 103 ± 20 MPa; while for the PEEK implant, it was 99 ± 11 MPa. In this case, no significant differences are observed (*p* > 0.05), with a confidence level of 95%.

The total deformation of the PMMA implant was also examined, where the mean and standard deviation of the analysis were 2.71 ± 0.22 mm; while for the PEEK implant, 2.30 ± 0.03 mm was obtained. In this case, significant differences were observed (*p* < 0.05), with a confidence level of 95%.

In Figure 13A, the Von Mises stress levels in the cranial bone are observed, resulting from the couplings of the PMMA and PEEK implants, respectively. Whereas, in Figure 13B, the total deformation of the cranial bone caused by the coupling of the implant is presented. All of the above under the application of a load “Force” 2000 N.

During the computational analysis, the effect of using PMMA and PEEK implants, respectively, coupled to the cranial bone, was examined to achieve the necessary protection of the internal structures and tissues, before a static load of 8000 N on the external surface of the implant. The mean and standard deviation of the Von Mises stress analysis for the cranial bone was 83 ± 7 MPa with PMMA. While with PEEK 59 ± 22 MPa was obtained. In this case, significant differences were observed (*p* < 0.05), with a confidence level of 95%.

The total deformation of the cranial bone when coupled with the PMMA implant was also examined. The values for the mean and standard deviation of the analysis were 0.213 ± 0.008 mm. For PEEK, in the same situation, 0.208 ± 0.009 mm was obtained. No significant differences are observed (*p* > 0.05), with a confidence level of 95%.

The analysis focused only on the case of the greater load because the safety of the personalized implant will depend on this action. The load of 2000 N simulates the incidence during the rest-activity; in such a situation, failures will not occur, since the stresses generated in the implant do not exceed the yield stress of either of the two materials [28,34].

The simulation results show that both devices (manufactured with PEEK and PMMA, respectively) present a similar resistance to the action of considered external loads. But the PMMA-based device suffers from greater deformation. However, for this second material, the fluence limit is not exceeded, that is, there is no risk of damage due to contact of the device with the brain mass. Furthermore, an additional option to increase the safety of the PMMA implant would be to control its thickness in such a way as to reduce deformation.

### 3.2. Post-Operative

**Figure 14:**
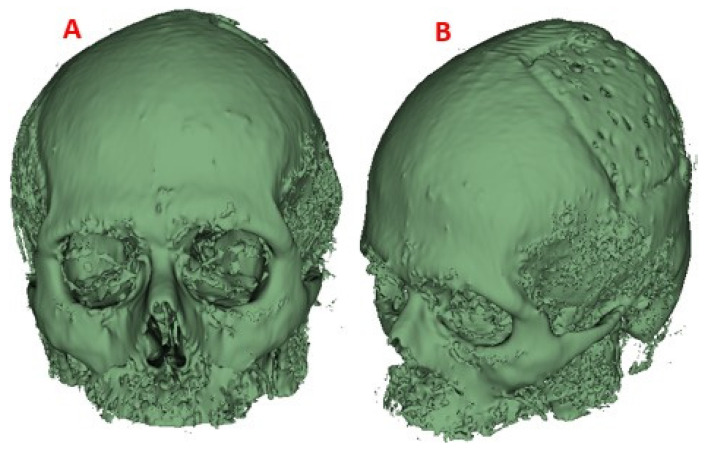
CT scan of the patient, with the custom PMMA implant, 14 days post-surgery: (**A**) Image of the skull with the PMMA implant attached, front view; (**B**) Image of the skull with the attached PMMA implant, 3D view.

The implant manufactured in PMMA was successfully placed in the patient. The postoperative evolution was satisfactory, there were no complications of any kind, and the person left the hospital facility 48 h after the intervention. In addition, for follow-up, a visit was scheduled 14 days after surgery. In Figure 14, the symmetry achieved with the cranial reconstruction is visible. There were also no difficulties with healing, the aesthetic and functional result was as expected and completely to the patient’s satisfaction.

#### Postoperative Follow-Up

**Figure 15:**
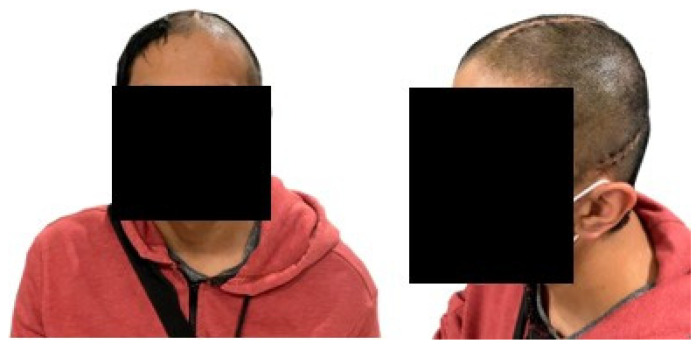
Patient benefited from the personalized PMMA implant.

Computer-aided design and additive manufacturing have proven to be effective tools in the design of custom implants. The results presented describe the entire custom manufacturing process, surgical planning, finite element analysis, and 3D manufacturing, which facilitated the performance of cranioplasty in a patient affected by skull bone cancer. The general procedure (fabrication of the implant and surgical planning) was carried out according to the proposed methodology and using open-source and commercial software for the segmentation, post-processing and mechanical design stages. The use of free software, whenever possible, was an advantageous factor considering the economic possibilities of the region where the methodology has been applied. In Figure 15, the final results of the entire process can be seen.

## 4. Discussion and Conclusions

Through the presentation of a real case, the usefulness of the proposed method for the analysis of clinical cases in the area of neurosurgery and reconstruction of cranial defects with a complex surgical approach was demonstrated. Through a finite element study, the level of Von Mises stresses and total deformations that take place in the coupling of the bone-implant system under static loads were determined, which is established as one more criterion for the design of medical devices for the cranioplasty treatment with biocompatible materials such as PMMA and PEEK.

Four computational analyses were developed under a static external load of 2000 N, which was applied to the upper left part of the implant, simulating an external action that this area of the head would receive during rest-activity; and a load of 8000 N, applied in the centre of the implant, simulating a static impact action. All of the above to determine the stress levels and total deformations of the implant-cranial bone system. In this investigation it was found that there are no significant differences between the PMMA personalized medical device and the PEEK one, concerning the total deformations caused by the load of 8000 N, considering the state of load as maximum. Significant differences were found in the Von Mises stress distribution.

The decision to know which device to use for cranioplasty treatment will depend on the magnitude of the damage and the patient’s financial availability. In Eastern European and Asian countries, PMMA is currently used as an alternative material for cranioplasty. In [35], is reported on the use of locally developed polylactic acid and polymethyl methacrylate moulds to perform cranioplasties for bone defects in technically demanding areas of the skull, while ensuring good aesthetic results and functional recovery. According to the authors, no surgical complications occurred in 14 patients. In addition, the subjective and objective evaluation revealed a significant improvement in the results. There were also no postoperative complications during a 6-month follow-up period, except in one patient who presented a late infection. Studies like this validate the use of acrylic materials as an alternative for cranioplasty treatment.

Late infection rates from the use of PMMA in custom-made bone implants are frequently increased by improper handling of sterilization protocols for manufactured devices, or by bacterial contamination during handling.

It has also happened that, in patients who have undergone successive surgery to remove and replace implants, the capillary tissue has lost healing properties, causing the decubitus effect and with it the proliferation of bacteria that contaminate the implanted device. Even so, the cranioplasty surgeries that have been performed in this area of Ecuador have had an approximate average cost of USD 450. In addition, the preoperative preparation with the anatomical test models has helped to reduce the duration of the interventions and the time the patient remains under the effects of anaesthesia, also reducing the risks of surgical complications. A similar scenario is described in [35,36]. As reported, the investment in a PMMA implant was USD 50.

The manufacturing process with PEEK has an approximate cost of USD 5000, inaccessible to the economy of many people. In the present study, however, the simulation results showed that both materials are equal in terms of security and structural integrity that they provide in the event of static load events: they do not exceed the yield point, so under the same demands, they are capable of responding without failure. Regarding the total deformations suffered, no significant differences were observed. In addition, the possible scope of the finite element method has been verified in terms of optimizing the structural properties of the implant to maximize resistance and durability and minimize weight and volume.

In some public and private hospitals in the Republic of Ecuador, a country located in the northwestern region of South America, the introduction of advanced medical technologies, such as 3D printing, has been fundamentally limited for economic reasons and a lack of investment in research and development. However, in a certain way, these limitations have been overcome by applying viable alternatives. For example, keeping PMMA as a feasible option in terms of biocompatibility and mechanical performance, and advantageous in economic terms. So, considering the above factors in terms of benefit-cost, an immediate alternative for progress could be that, when the solution in terms of manufacturing material for a customized implant be PMMA, prior to device placement, a test of allergy (for example, a patch type test) to try to predict the behaviour of the prosthesis once in contact with the body of the patient who will receive it.

In three health institutions in the region, since July 2020, five surgeries were performed that required customized bone replacement (the last one made in September 2022). Three cranioplasties, one clavicular implant and one for the sternum-clavicle. All medical devices placed in these patients were manufactured with PMMA. Follow-up has been maintained on each of them up to the present, and in none of the cases has there been any complication. These surgical procedures have been performed under the protection of the rules of the National Agency for Health Regulation, Control and Surveillance (ARCSA-acronym in Spanish), RESOLUTION No. ARCSADE0262016YMIH, Article 20, items (a), (b), (c) and (d).

## Figures and Tables

**Table 1:** Material properties of components used in the simulation of the assembly model 3D.

Parameter	Cranial Cortical Bone	PMMA	PEEK
Young’s modulus	*Eb* = 15,000 MPa	*EPMMA* = 3000 MPa	*EPEEK* = 3600 MPa
Poisson’s ratio	*µb* = 0.3	*µPMMA* = 0.38	*µPEEK* = 0.39
Ultimate tensile	*σu*,*b* = 130 MPa	—–	*σu*,*PEEK* = 172 MPa
Yield strength	—–	*σy*,*PMMA* = 72 MPa	*σy*,*PEEK* = 90 MPa
Reference	[32]	[33]	[33]

**Table 2:** FDM additive manufacturing characteristics and parameters.

Characteristics and Manufacturing Parameters	Fused Deposition Modelling
Company and model	Creality CR-X Pro (2019 Updated)
Maximum build envelope	300 × 300 × 400 mm^3^
Nozzle diameter	0.4 mm
Positioning resolution (X/Y/Z)	1.25 µm/1.25 µm/1 µm
Selected layer thickness	0.10 mm
Printed filament line width	0.4 mm

**Table 3:** Von Mises stresses and total deformation of the structures *.

Structure	Material	Von Mises Stress [MPa]	Total Deformation [mm]	Load State [N]
Skull	Cortical bone	40 ± 10	0.080 ± 0.001	2000
Implant	PMMA	40 ± 7	0.56 ± 0.02	2000
Skull	Cortical bone	36 ± 8	0.073 ± 0.001	2000
Implant	PEEK	44 ± 5	0.42 ± 0.08	2000
Skull	Cortical bone	83 ± 7	0.210 ± 0.008	8000
Implant	PMMA	103 ± 20	2.71 ± 0.21	8000
Skull	Cortical bone	59 ± 22	0.21 ± 0.01	8000
Implant	PEEK	99 ± 11	2.30 ± 0.03	8000

* Each load state was applied to the external surface of the implant, at the same point. The effort with the deformation generated in the cranial structure is the result of the coupling and transfer of the load on the implant.

## Data Availability

Data included in article/referenced in the article.
